# A Case of Methicillin-Sensitive Staphylococcus aureus Septic Arthritis Causing Septic Pulmonary Emboli in a Healthy Young Adult

**DOI:** 10.7759/cureus.98448

**Published:** 2025-12-04

**Authors:** Ahmed H Abdelfattah

**Affiliations:** 1 Internal Medicine, University of Kentucky College of Medicine, Lexington, USA

**Keywords:** deep vein thrombosis (dvt), immunocompetent individuals, mssa bacteremia, pulmonary septic emboli, septic arthritis, septic emboli

## Abstract

Disseminated *Staphylococcus aureus* (*S. aureus*) infection in healthy adults is uncommon and usually linked to endocarditis, intravenous (IV) drug use, or immunocompromise. I describe a 21-year-old man who presented with acute right hip pain, fever, and difficulty bearing weight. Evaluation revealed right hip septic arthritis complicated by bilateral septic pulmonary emboli (SPE) and right femoral vein thrombosis. Transthoracic and transesophageal echocardiograms showed no evidence of endocarditis. Blood and joint cultures grew methicillin-sensitive *S. aureus* (MSSA). He completed six weeks of continuous-infusion intravenous cefazolin with good clinical recovery. This case demonstrates that disseminated MSSA infection with septic emboli can arise in immunocompetent adults without endocarditis and emphasizes the need to consider septic arthritis as a potential primary source.

## Introduction

*Staphylococcus aureus* bacteremia (SAB) remains a leading cause of serious bloodstream infections and is associated with significant morbidity from metastatic complications, including endocarditis, osteomyelitis, and septic arthritis. Dissemination without an identifiable cardiac source, however, is uncommon and diagnostically challenging [1]. Septic pulmonary emboli (SPE) from deep tissue infections are increasingly reported in children with community-acquired staph bacteremia but remain rarely described in adults [1,2]. Classic predisposing factors for SPE include intravenous (IV) drug use, endocarditis, indwelling catheters, and septic thrombophlebitis [2]; however, SPE in otherwise healthy adults without these risk factors is rare and diagnostically challenging [1]. I report a rare case of methicillin-sensitive *Staphylococcus aureus* (MSSA) septic arthritis complicated by deep vein thrombosis (DVT) and bilateral SPE in a young adult with no evidence of endocarditis, emphasizing that severe disseminated infection can occur even in immunocompetent individuals.

## Case presentation

A 21-year-old man with a history of migraines and nicotine dependence presented with a four-day history of worsening right hip and groin pain that began after a routine workday. The pain rapidly progressed to the point that he could no longer bear weight, and he subsequently developed right-sided chest pain, dyspnea, and tachycardia. He denied trauma, recent travel, illicit drug use, or prior systemic infections.

**Figure 1:**
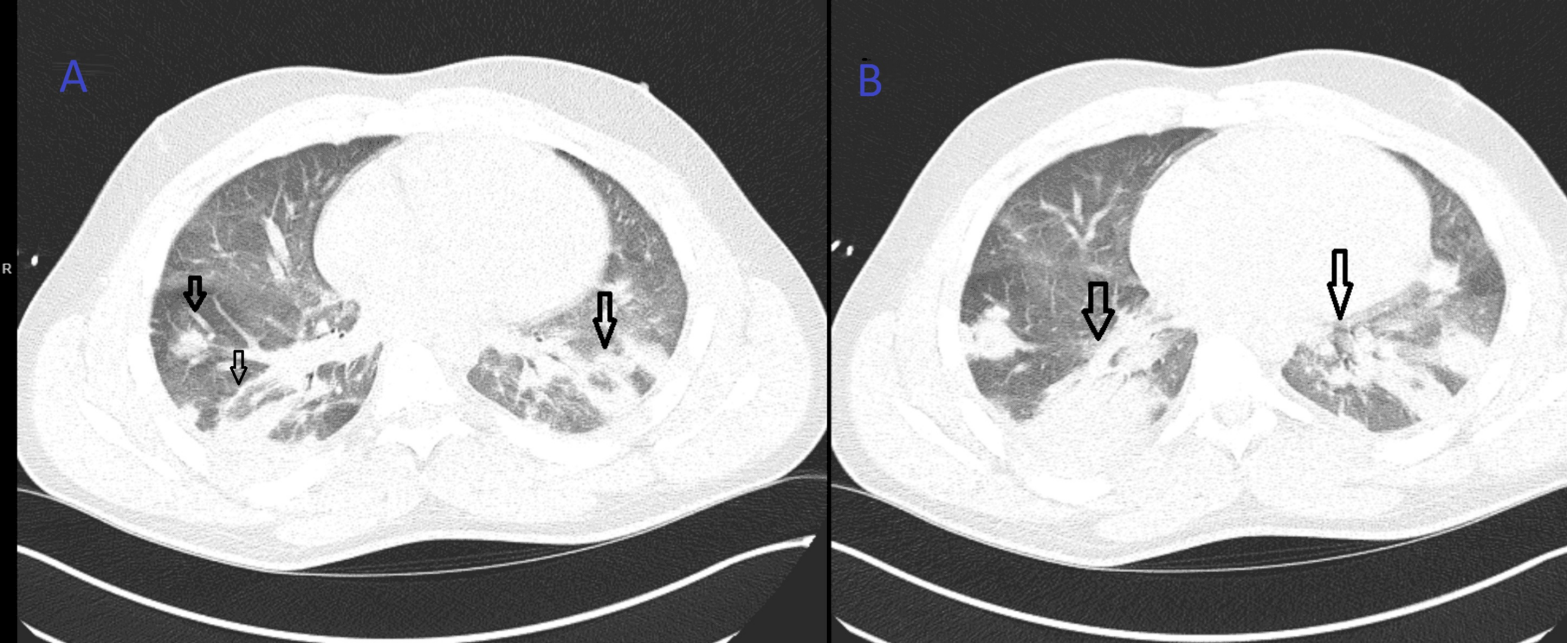
Septic pulmonary emboli, illustrating a multifocal and occasionally cavitating pattern (A) Axial CT showing bilateral patchy ground-glass opacities and variably sized nodules, some cavitating (black arrows). (B) Axial CT showing consolidation and persistent ground-glass opacities (black arrows).

Initial evaluation at an outside facility revealed a right hip effusion with surrounding soft tissue inflammation concerning for septic arthritis and possible myositis. CT of the chest demonstrated numerous bilateral peripheral nodules consistent with septic pulmonary emboli, as shown in Figure 1.

Bedside echocardiography suggested possible endocarditis, and blood cultures grew methicillin-sensitive *Staphylococcus aureus* (MSSA). He was transferred to our institution for further management.

**Figure 2:**
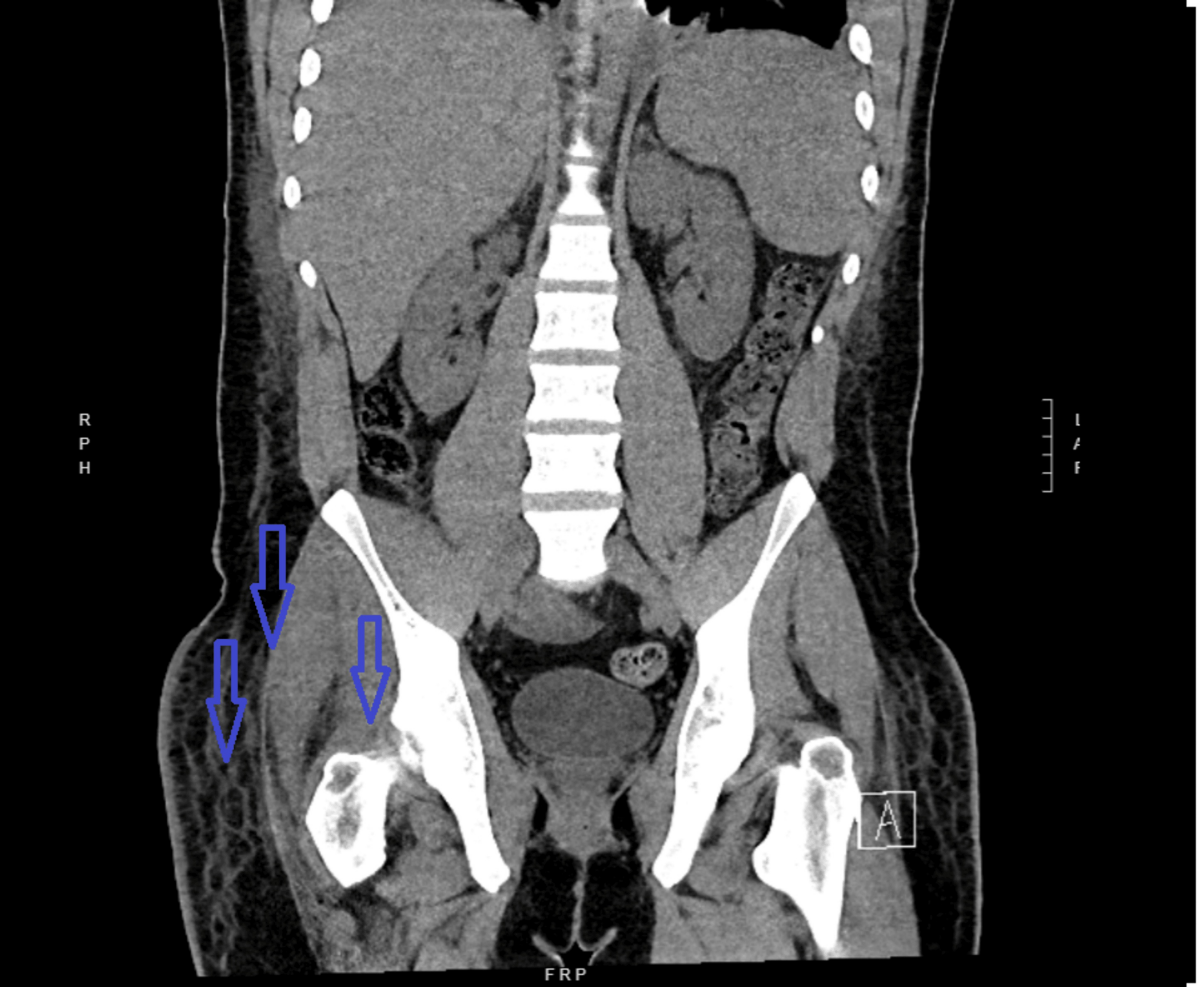
Right hip joint effusion Blue arrows pointing to the effusion and fat stranding concerning for septic arthritis.

On arrival, he was mildly tachycardic and required supplemental oxygen, later escalating to high-flow nasal cannula due to increasing hypoxia. Physical examination revealed significant pain with movement of the right hip. Laboratory studies showed leukocytosis, elevated inflammatory markers of C-reactive protein, and mild acute kidney injury. CT of the abdomen and pelvis was done due to worsening pain in the right hip joint, which showed right hip joint effusion with a surrounding soft tissue stranding concerning for septic arthritis, as shown in Figure 2.

Right hip aspiration produced purulent fluid that subsequently grew MSSA. Blood cultures showed MSSA bacteremia as well. Transthoracic and transesophageal echocardiograms were done and showed no valvular vegetations. Right lower extremity swelling progressed during hospitalization; Doppler ultrasound revealed an acute right common femoral vein thrombosis. CT and MRI of the abdomen and spine demonstrated no additional abscesses or evidence of osteomyelitis.

The patient underwent operative irrigation and debridement of the right hip with clinical improvement. Antibiotic therapy was narrowed to intravenous cefazolin (6 g/day via continuous infusion) based on infectious disease recommendations. Therapeutic anticoagulation was initiated with enoxaparin and transitioned to apixaban. His respiratory status gradually improved, inflammatory markers declined, and repeat blood cultures remained negative.

He was discharged in stable condition to complete a six-week course of intravenous cefazolin via outpatient parenteral antimicrobial therapy (OPAT).

## Discussion

Disseminated MSSA infection in a young, immunocompetent host is uncommon. Most reported cases occur in individuals with identifiable risk factors such as intravenous drug use, indwelling vascular catheters, or underlying immunosuppression [1]. Although soft tissue infection is a recognized source of septic pulmonary emboli (SPE) [2], this manifestation is rare in adults and is infrequently described in the literature [3].

My patient presented with MSSA septic arthritis complicated by femoral vein thrombosis and septic pulmonary emboli, yet without evidence of right-sided endocarditis. This constellation suggests septic arthritis as the likely source of systemic dissemination, emphasizing that peripheral deep tissue infections can lead to life-threatening metastatic complications even in otherwise healthy individuals.

Comparable cases have been reported. One study described an immunocompetent patient who developed MRSA bacteremia and SPE arising from a peripheral venous catheter despite no intravenous drug use or other traditional risk factors. Transesophageal echocardiography revealed no right-sided endocarditis, highlighting that severe septic embolic complications can arise from peripheral catheter-associated infections alone [4]. Another published case involved extended-spectrum beta-lactamase (ESBL), producing *Klebsiella pneumoniae* as the causative pathogen, further demonstrating that SPE may result from a variety of nontraditional sources [5].

This case also underscores the diagnostic challenges of SPE. Early clinical recognition is difficult because symptoms, such as dyspnea, pleuritic chest pain, and hypoxia, can be nonspecific. Imaging studies, particularly chest CT, are essential to identify the characteristic peripheral nodules, some with cavitation, that distinguish septic pulmonary emboli from other pulmonary pathologies. Moreover, prompt blood cultures, joint aspiration, and echocardiography are crucial for identifying the causative organism and ruling out endocarditis.

Therapeutic considerations in SPE include targeted intravenous antibiotics, surgical management of the primary source of infection, and anticoagulation in cases complicated by septic thrombophlebitis or deep vein thrombosis. In this case, early operative debridement of the hip, tailored IV cefazolin therapy, and anticoagulation resulted in clinical improvement and resolution of bacteremia. This aligns with prior reports suggesting that multimodal therapy, combining source control, antibiotics, and anticoagulation when indicated, is key to favorable outcomes.

Finally, this case emphasizes that clinicians should maintain a high index of suspicion for SPE in patients with musculoskeletal infections, even when classical risk factors are absent. Awareness of this rare but serious complication may facilitate earlier diagnosis, reduce morbidity, and improve survival.

## Conclusions

This case highlights a rare presentation of MSSA septic arthritis complicated by femoral vein thrombosis and SPE in a young, immunocompetent adult without endocarditis. It underscores the importance of maintaining a high index of suspicion for septic pulmonary embolism in patients with soft tissue or joint infections, even in the absence of traditional risk factors such as intravenous drug use or indwelling catheters. Early recognition, prompt imaging, timely surgical intervention, and appropriate antimicrobial therapy are crucial to preventing morbidity and ensuring favorable outcomes in such uncommon but potentially severe infections.
